# Cardiac Autonomic and Endothelial Function in Acute Lymphoblastic Leukaemia Patients Immediately After Chemotherapy and at the Three-Month Follow-up

**DOI:** 10.7759/cureus.55108

**Published:** 2024-02-27

**Authors:** Sameer Sameer, Prabhu N, Saranya Kuppusamy, Prashant S Adole, Smita Kayal

**Affiliations:** 1 Physiology, Jawaharlal Institute of Postgraduate Medical Education and Research, Puducherry, IND; 2 Biochemistry, Jawaharlal Institute of Postgraduate Medical Education and Research, Puducherry, IND; 3 Medical Oncology, Regional Cancer Center, Jawaharlal Institute of Postgraduate Medical Education and Research, Puducherry, IND

**Keywords:** chemotherapy-related cardiac dysfunction (ctrcd), inflammation, endothelial damage, cardiovascular autonomic function, acute lymphoblastic leukemia

## Abstract

Introduction: Acute lymphoblastic leukemia (ALL) is a malignant uncontrolled overproduction of immature lymphoid cells in blood and bone marrow. The primary treatment of ALL is chemotherapy. Chemotherapy can have myriad systemic side effects, notably cardiovascular derangement. Autonomic derangement occurrence in cancer patients signifies cardiovascular risk in them and is a determinant of cardiovascular morbidity and mortality. Elevated soluble vascular cell adhesion molecule-1 (sVCAM-1) and soluble intercellular adhesion molecule-1 (sICAM-1) levels implicated in the regulation of inflammation indicate endothelial dysfunction. High levels of high-sensitivity C-reactive protein (hsCRP) can be indicative of low-grade inflammation. Hence, in this study the cardiac autonomic function and endothelial and inflammatory biomarker levels in adult patients with ALL were assessed immediately and three months after chemotherapy.

Methods: In this longitudinal study, 30 ALL patients (23 males, seven females) aged between 18 to 50 years, who had completed chemotherapy regimens, and 30 age and gender-matched healthy participants (controls) were recruited. Cardiac autonomic function tests (short-term heart rate variability (HRV), 30:15 ratio, synaptic excitation and inhibition (E/I) ratio, diastolic blood pressure (DBP) response to isometric hand grip), endothelial markers (sVCAM-1 and sICAM-1), and inflammatory marker (hsCRP) were assessed immediately and at three months after chemotherapy.

Results: Magnitudes of time domain and frequency domain indices, conventional autonomic function test indices, and biomarkers were deranged in ALL patients immediately after chemotherapy. After three months, cardiac autonomic function parameters were found to improve in the form of increased root mean square of successive differences between normal heartbeats (RMSSD), standard deviation of the interbeat intervals of normal sinus beats (SDNN), total power, high-frequency (HF)nu, and decreased low-frequency(LF)nu & LF-HF ratio. Endothelial (sVCAM-1) and inflammatory markers (hsCRP) were lower in the patient group as compared to the controls immediately after chemotherapy. Three months after chemotherapy, the levels of endothelial and inflammatory markers did not show much change.

Conclusion: In this study, we found ALL patients showed higher sympathetic drive, decreased parasympathetic modulation, and sympathovagal imbalance immediately after chemotherapy as compared to the controls, indicating cardiovascular risk. After three months, improvement in cardiovascular autonomic function was observed. ALL itself is a state of inflammation with elevated endothelial and inflammatory markers; thus, the decreased endothelial and inflammatory markers could be attributed to the immediate effect of chemotherapy.

## Introduction

Acute lymphoblastic leukemia (ALL) is heralded by the unrestrained proliferation of lymphoid progenitor cells in the bloodstream, bone marrow, and various tissues. In ALL, "acute" denotes the existence of immature circulating lymphocytes known as "blasts".

ALL stands as the most prevalent form of childhood cancer, with around 75% of leukemia cases diagnosed in individuals under the age of 20 being attributed to ALL. However, ALL can be diagnosed at any age [1]. ALL treatment has been a remarkable success story in pediatric oncology. However, in the case of patients older than 40 years, outcomes tend to be less favorable compared to children. Swift and accurate diagnosis, coupled with aggressive treatment measures such as chemotherapy and radiation therapy is vital for effectively managing this aggressive form of leukemia. Ongoing research and medical advancements strive to improve outcomes for patients with ALL.

The primary treatment of ALL is chemotherapy. Chemotherapy for treating ALL typically involves three distinct phases: induction, consolidation, and maintenance. In the induction phase (four weeks) a complete remission is aimed for with the help of drugs usually vincristine and corticosteroids [2]. The consolidation phase consists of postinduction chemotherapy (11 months or sometimes more). The most commonly used schema is the Berlin-Frankfurt-Münster (BFM) backbone which includes cyclophosphamide and intrathecal therapy [3]. The maintenance phase (≥ 6 months) starts after the consolidation phase, and in this phase methotrexate (MTX) and mercaptopurine [4] are used. 

Chemotherapy can have myriad systemic side effects. Vincristine manifests severe systemic side effects, encompassing bone marrow depression, electrolyte imbalance, peripheral neuropathy, and gastrointestinal disorders. Notably, recent research findings underscore the emerging concern of possible cardiovascular damage during the administration of this treatment [5]. Cyclophosphamide can cause substantial cardiac toxicity, including fatal myocarditis, particularly at higher doses [6].

ALL survivors have a greater preponderance of developing cardiac disease compared to healthy controls. Christoffersen et al. reported that over one-third of childhood ALL survivors develop cardiac autonomic dysfunction (CAD) in the form of lower exercise capacity, which is higher compared to age, gender, race, ethnicity, drug-matched controls that predispose them to poor exercise performance, thereby enhancing the risk of cardiovascular comorbidities like the enhanced risk of arrhythmia and sudden cardiac death [7]. Kamath et al. demonstrated reduced resting state heart rate variability (HRV) abnormalities among childhood ALL survivors [8]. Autonomic derangement in cancer is believed to be an early predictor of the occurrence of cardiovascular risk and is a determinant of cardiovascular morbidity and mortality. Cardiovascular autonomic function assessment, which is a non-invasive test, has proven to be effective in cardiovascular risk prediction and stratification.

Endothelial markers such as soluble intercellular adhesion molecule-1 (sICAM-1) and soluble vascular cell adhesion molecule-1 (sVCAM-1) are involved in inflammatory regulation [9]. ICAM-1 is a ligand for LFA-1 (integrin), a receptor found on leukocytes; it is important in facilitating leukocyte endothelial transmigration. The VCAM-1 protein mediates the adhesion of lymphocytes, monocytes, eosinophils, and basophils to vascular endothelium. These adhesion molecules cause the adhesion of immune cells to the endothelium, which is essential in the initiation and progression of vascular inflammation. Elevated sICAM-1 and sVCAM-1 indicate endothelial damage, which is crucial in the onset of atherosclerosis and other cardiovascular diseases (CVDs) [10]. Endothelial dysfunction (ED) is a prevalent characteristic in the initial complications of hemato-oncologic therapy. Despite ongoing refinement of treatment protocols, it remains a substantial contributor to morbidity and mortality. Hence, these therapies may be used as biomarkers to assess cardiovascular risk and monitor the progression of CVD.

High-sensitivity C-reactive protein (hs-CRP), has always been related to adverse cardiovascular events. Research suggests that high levels of hs-CRP can be indicative of low-grade inflammation within the body and have been linked to atherosclerosis and coronary artery disease [11].

Therefore, considering the cardiovascular derangement, it would be prudent to investigate the cardiovascular risk status in patients with ALL on completion of chemotherapy. Considering the paucity of studies in adult patients with ALL on chemotherapy, this study intends to evaluate the cardiac autonomic function and endothelial and inflammatory biomarker levels in adult patients with ALL, immediately after completion of chemotherapy and at three months after completion of chemotherapy.

## Materials and methods

Study design

This longitudinal quantitative study was carried out at the Department of Physiology, Jawaharlal Institute of Postgraduate Medical Education and Research (JIPMER), Puducherry, India, after obtaining the necessary approval of the Institute Ethics Committee for Observational Studies (Human) JIPMER (approval number: JIP/IEC/2021/236).

In this study, 30 ALL patients (23 males, seven females) who had undergone a similar prescribed course of chemotherapy regimen were recruited as the study group from the Medical Oncology outpatient department, JIPMER, India. Patients with known CVD prior to chemotherapy, diabetes mellitus, renal failure, heart failure, and hypertension were excluded. Appropriate age and gender-matched 30 healthy volunteers (21 males, nine females) were recruited as controls. Volunteers with known CVD, diabetes mellitus, renal failure, heart failure, and hypertension were excluded from the study. Since ovarian hormones influence autonomic functions and HRV, female participants (in both groups) were recruited during the follicular phase of the menstrual cycle [12-14]. The study participants were followed for three months and 26 ALL patients were able to participate in the study at the end of three months because two male patients expired and two male patients went into relapse.

Procedure

The participants reported to the Autonomic Function Testing Lab, after light breakfast, in loose clothing, and were asked to refrain from tea, coffee, nicotine, and alcohol 24 hours before the test. Written informed consent was obtained from all participants, after a detailed explanation of the study procedure. The parameters that were recorded, both immediately and three months post chemotherapy, are given below.

Parameters Measured

Anthropometry: Participants’ height was measured using a wall-mounted stadiometer (BHH6, EASYCARE GmbH, Mumbai, India) and weight was recorded using an electronic weighing machine (MS 4900, Charder Electronics Co. Ltd, Taiwan). Quetelet's index was used to calculate body mass index (BMI). Waist circumference was taken at the narrowest point between the tenth rib and the iliac crest. Hip circumference was measured as the maximum gluteal circumference. Additionally, the waist-to-hip ratio was computed.

Basal cardiovascular parameters: Following 10 minutes of rest in the supine posture, basal heart rate (BHR) and blood pressure (BP) (systolic BP (SBP) and diastolic BP (DBP)) were measured with a digital BP monitor (Omron, HEM - 8712, Omron Healthcare Co. Ltd, Kyoto, Japan).

Cardiovascular autonomic function tests: (a) Short-term HRV: Short-term HRV assessment was done following the guidelines of the Taskforce of the European Society of Cardiology and the North American Society of Pacing and Electrophysiology, with a five-minute resting lead II ECG. BIOPAC-MP150 system (Biopac Systems Inc., Goleta, California, United States) along with AcqKnowledge software version 4.2 was used for data acquisition of lead II ECG. HRV analysis was performed using Kubios HRV standard software, version 3.2 (Kubios Oy, Kuopio, Finland). Time domain indices (root mean square of successive differences between normal heartbeats (RMSSD), standard deviation of the interbeat intervals of normal sinus beats (SDNN), NN50, pNN50) and frequency domain indices (very low frequency (VLF) (ms2), low frequency (LF) (ms2), high frequency (HF) (ms2), Total Power (ms2), LF (nu), HF (nu), and LF/HF ratio) were computed.

(b) 30:15 ratio: After five minutes of supine rest, subjects stood actively for five minutes, during which lead II ECG, heart rate (HR), and BP were measured. The 30:15 ratio was computed as the ratio of the longest RR interval at the 30th beat to the shortest RR interval at the 15th beat.

(c) E: I ratio: The participants performed deep breathing at the rate of six cycles/minute, with each breath over 10 seconds (5 seconds each for inspiration and expiration). Continuous lead II ECG recording was conducted during the maneuver. The E:I ratio is computed as the ratio of the longest RR interval during expiration to the shortest RR interval during inspiration averaged over six cycles of respiration.

(d) BP response to isometric handgrip (IHG) test: The DBP was measured during sustained IHG over three minutes, maintained at one-third of maximum voluntary capacity (MVC). The magnitude of change in DBP during the maneuver (∆DBPIHG, given as the difference between the highest DBP reached during the sustained handgrip and resting DBP) was measured.

Endothelial and inflammatory marker: 5 ml of venous blood was collected for biochemical analysis. Endothelial biomarkers (sVCAM-1 and sICAM-1) were estimated by enzyme-linked immunosorbent assay (ELISA) (Krishgen Biosystems, Cerritos, California, United States). Inflammatory biomarker hs-CRP was estimated by ELISA (Calbiotech Inc., Cordell Ct., El Cajon, California, United States).

Sample size estimation

The sample size was estimated based on the estimation procedure for the comparison of two independent means, estimated using PS: Power and Sample Size Calculation version 3.1.6 (Vanderbilt University Medical Center, Nashville, Tennessee, United States). The sample size was estimated based on the effect of chemotherapy on the LF:HF ratio, with a mean difference of 0.3 between the groups with a standard deviation of 0.43. The estimated sample size was 27 in each group and the corrected sample size was 30 in each group, considering 10% dropout at a 5% level of significance and 90% power [8].

Statistical analysis

Data analysis was carried out using IBM SPSS Statistics for Windows, Version 19.0 (Released 2010; IBM Corp., Armonk, New York, United States). All the continuous variables such as age, weight, height, HRV indices, etc. were summarized as mean with standard deviation. The continuous variables were assessed for normality using Kolmogorov-Smirnov test. The inter-group differences in parameters between controls and the ALL patients were compared using independent student’s t-test. The intra-group differences between ALL patients and their follow-up after three months were compared by paired t-test. The biochemical parameters between control and ALL patients were compared by Mann-Whitney U test and between ALL patients and their follow-up after three months were compared by Wilcoxon signed-ranks test. All the analyses were carried out at a 5% level of significance (95% confidence interval) and a P value < 0.05 was considered statistically significant.

## Results

Anthropometric and basal cardiovascular parameters

Table 1 compares anthropometric and basal cardiovascular parameters between the controls and ALL patients immediately after chemotherapy. There was no difference in the anthropometric parameters between the two groups. Among basal cardiovascular parameters, a significantly lower diastolic BP (p=0.013) was observed; SBP and BHR were lower but not significant. Table 2 shows the comparison of anthropometric and basal cardiovascular parameters immediately and at three months after chemotherapy. No significant change was observed in anthropometric parameters three months after chemotherapy. A slight decrease in systolic BP and an increase in mean HR were observed (Table 2). 

**Table 1:** Comparison of anthropometric measurements between control group and ALL patients immediately after chemotherapy Analysis done by independent student t-test; p-value less than 0.05 are considered statistically significant. ALL: acute lymphoblastic leukemia

Variable	Controls, Mean (SD) (n=30)	ALL patients Immediately after chemotherapy, Mean (SD) (n=30)	p-value
Height (cm)	1.70 (0.08)	1.67 (0.08)	0.210
Weight (kg)	70.63 (15.96)	65.55 (13.70)	0.191
BMI (kg/m^2^)	24.41 (5.11)	23.52 (5.20)	0.507
Waist Circumference (cm)	87.57 (13.54)	88.50 (12.27)	0.781
Hip Circumference (cm)	97.60 (9.93)	98.67 (11.03)	0.695
Systolic Blood Pressure (mmHg)	113.47 (10.32)	107.87 (12.84)	0.068
Diastolic Blood Pressure (mmHg)	71.70 (8.99)	64.97 (11.29)	0.013*
Basal Heart Rate (beats/min)	78.05 (6.99)	74.78 (6.85)	0.073

**Table 2:** Comparison of anthropometric measurements of ALL patients immediately after chemotherapy and three months after chemotherapy Analysis done by independent student t-test; p-value less than 0.05 is considered statistically significant. ALL: acute lymphoblastic leukemia

Variable	Immediately after chemotherapy, Mean (SD) (n=26)	Three months after chemotherapy, Mean (SD) (n=26)	p-value
Height (cm)	1.66 (0.09)	1.66 (0.10)	0.553
Weight (kg)	63.56 (13.31)	64.68 (14.07)	0.054
BMI (kg/m^2^)	23.00 (5.28)	23.42 (5.22)	0.235
Waist Circumference (cm)	85.84 (11.19)	85.44 (12.51)	0.763
Hip Circumference (cm)	95.72 (9.10)	96.36 (10.33)	0.636
Systolic Blood Pressure (mmHg)	107.96 (12.72)	105.77 (13.92)	0.506
Diastolic Blood Pressure (mmHg)	64.96 (11.62)	64.65 (7.12)	0.893
Basal Heart Rate (beats/min)	74.69 (7.30)	76.03 (9.80)	0.630

Short-term HRV analysis

Time Domain Indices

Table 3 shows that magnitudes of SDNN, RMSSD, NN50, and pNN50 were lower in ALL patients as compared to the controls immediately after chemotherapy in which RMSSD was found statistically significant (p=0.023). Table 4 shows that magnitudes of SDNN, RMSSD, NN50, and pNN50 increased in ALL patients three months after chemotherapy, although not significant.

**Table 3:** Comparison of cardiac autonomic function test parameters between control group and ALL patients immediately after chemotherapy Analysis done by independent student t-test; p-value less than 0.05 is considered statistically significant. RMSSD: root mean square of squared differences of successive normal to normal intervals; SDNN: standard deviation of normal to normal interval; NN50: the number of interval differences of successive NN intervals greater than 50 ms; pNN50: the proportion derived by dividing NN50 by the total number of NN intervals; LF nu: low-frequency component expressed as a normalized unit; HF nu: high-frequency component expressed as a normalized unit; LF-HF ratio: ratio of low-frequency power to high-frequency power; 30:15 ratio: ratio between maximum RR interval at 30th beat and minimum RR interval at 15th beat; E:I ratio: ratio of longest RR interval during expiration to shortest RR interval during inspiration averaged over six cycles of respiration; DDBPihg: difference in diastolic blood pressure between supine and Isometric handgrip; ALL: acute lymphoblastic leukemia

Variable	Control group, Mean (SD) (n=30)	ALL patients Immediately after chemotherapy, Mean (SD) (n=30)	p-value
Time domain indices			
RMSSD (ms)	37.46 (16.66)	27.77 (15.40)	0.023
SDNN (ms)	43.00 (17.64)	39.32 (19.63)	0.448
NN50	53.03 (47.42)	30.26 (43.03)	0.056
pNN50 (%)	15.40 (12.98)	9.94 (13.94)	0.122
Frequency domain indices			
LF (ms^2^)	616.60 (456.12)	395.27 (430.769)	0.058
HF (ms^2^)	886.67 (800.10)	349.17 (434.79)	0.002
LF nu	43.12 (10.80)	57.96 (15.23)	<0.001
HF nu	56.87 (10.80)	42.03 (15.23)	<0.001
Total Power (ms^2^)	1935.03 (1479.49)	1156.47 (965.23)	0.019
LF-HF ratio	0.83 (0.45)	1.86 (1.57)	0.002
Cardiac autonomic reactivity tests			
30:15 ratio	1.08 (0.43)	0.96 (0.49)	0.293
E:I ratio	1.44 (0.30)	1.36 (0.37)	0.354
DDBP_ihg_ (mm Hg)	13.97 (6.8)	17.47 (12.66)	0.189

**Table 4:** Comparison of cardiac autonomic function test parameters of ALL patients immediately after chemotherapy and three months after chemotherapy Analysis done by independent student t-test; p-value less than 0.05 is considered statistically significant. RMSSD: root mean square of squared differences of successive normal to normal intervals; SDNN: standard deviation of normal to normal interval; NN50: the number of interval differences of successive NN intervals greater than 50 ms; pNN50: the proportion derived by dividing NN50 by the total number of NN intervals; LF nu: low-frequency component expressed as normalized unit; HF nu: high-frequency component expressed as normalized unit; LF-HF ratio: ratio of low-frequency power to high-frequency power; 30:15 ratio: ratio between maximum RR interval at 30th beat and minimum RR interval at 15th beat; E:I ratio: ratio of longest RR interval during expiration to shortest RR interval during inspiration averaged over six cycles of respiration; DDBPihg: difference in diastolic blood pressure between supine and isometric handgrip; ALL: acute lymphoblastic leukemia

Variable	Immediately after chemotherapy, Mean (SD) (n=26)	Three months after chemotherapy, Mean (SD) (n=26)	p-value
Time domain indices			
RMSSD (ms)	28.86 (16.28)	33.64 (17.40)	0.352
SDNN (ms)	40.73 (20.55)	41.66 (15.87)	0.837
NN50	33.53 (45.41)	38.83 (34.80)	0.614
pNN50 (%)	11.21 (14.58)	12.21 (12.82)	0.811
Frequency domain indices			
LF (ms^2^)	442.04 (455.64)	453.38 (292.85)	0.773
HF (ms^2^)	384.92 (456.80)	593 (683.34)	0.238
LF nu	57.9 (15.23)	53.2 (18.8)	0.509
HF nu	42 (15.2)	46.76 (18.8)	0.509
Total Power (ms^2^)	1231.69 (1013.07)	1469 (1149.32)	0.461
LF-HF ratio	1.86 (1.57)	1.67 (1.48)	0.758
Cardiac autonomic reactivity tests			
30:15 ratio	0.92 (0.52)	1.06 (0.14)	0.201
E:I ratio	1.36 (0.39)	1.38 (0.35)	0.858
DDBP_ihg_ (mm Hg)	16.88 (13.49)	14.35 (7.48)	0.411

Frequency Domain Indices

HF (p=0.002), HF nu (p=<0.001), and total power (p=0.019) were significantly lower and LF nu (p=<0.001) and LF-HF ratio (p=0.002) were significantly higher immediately after chemotherapy compared to controls (Table 3). After three months, LF, HF, HF nu, and total power increased. LF nu and LF-HF ratio decreased (Table 4).

Cardiac Autonomic Reactivity Tests

30:15 ratio and E:I ratio were lower and DDBPihg was higher in ALL patients immediately after chemotherapy as compared to controls (Table 3). After three months of chemotherapy, the 30:15 ratio and E:I ratio increased and DDBPihg was found to be reduced but it does not show statistical significance (Table 4).

Biochemical Parameters

sVCAM-1 was observed to be significantly lower in ALL patients immediately after chemotherapy compared to the controls (Table 5). However, sICAM-1 was observed to be higher and hs-CRP was observed to be lower. sICAM-1 decreased significantly in ALL patients three months after chemotherapy while sVAM-1 and hs-CRP increased slightly (Table 6).

**Table 5:** Comparison of biochemical parameters between control group and ALL patients immediately after chemotherapy Analysis done by Mann-Whitney U test; p-value less than 0.05 is considered statistically significant. sVCAM-1: soluble vascular cell adhesion molecule-1; sICAM-1: soluble intercellular adhesion molecule-1; hsCRP: high-sensitivity C-reactive protein; ALL: acute lymphoblastic leukemia

Variable	Control group, Median (IQR) (n=30)	ALL patients immediately after chemotherapy, Median (IQR) (n=30)	p-value
sVCAM-1 (pg/mL)	221 (186.6,264.2)	165.2 (153.9,173.2)	<0.001
sICAM-1 (ng/mL)	355.2 (201.7,549.2)	424 (344.7,564.6)	0.124
Hs-CRP (mg/L)	1.49 (0.5,4.1)	1.22 (0.6,2.9)	0.663

**Table 6:** Comparison of biochemical parameters of ALL patients immediately after chemotherapy and three months after chemotherapy Analysis done by Mann-Whitney U test; p-value less than 0.05 is considered statistically significant. sVCAM-1: soluble vascular cell adhesion molecule-1, sICAM-1: soluble intercellular adhesion molecule-1; hsCRP: high-sensitivity C-reactive protein; ALL: acute lymphoblastic leukemia

Variable	Immediately after chemotherapy, Median (IQR) (n=26)	Three months after chemotherapy, Median (IQR) (n=26)	p-value
sVCAM-1 (pg/mL)	161 (153.6,171.4)	164.72 (155.3,169.5)	0.620
sICAM-1 (ng/mL)	405.8 (332.8,564.6)	358 (341.9,409.2)	0.030
Hs-CRP (mg/L)	1.2 (0.6,2.95)	1.67 (0.9,2.7)	0.990

## Discussion

Although chemotherapy for ALL has improved significantly in recent years, it is limited by the risk of cardiotoxicity. Chemotherapy-induced cardiotoxicity is a critical adverse reaction, significantly elevating both morbidity and mortality rates. Furthermore, cardiac alterations are often subclinical, with manifestations that can emerge in the early, late, or very late stages. Studies regarding the intricate mechanism of cardiac dysfunction due to chemotherapy in adult ALL patients are scarce. Based on previous studies that have reported decreased HRV in childhood ALL survivors on chemotherapy [15], detailed cardiac autonomic function and endothelial and inflammatory markers in adult ALL patients have been evaluated in the current study immediately and three months after chemotherapy.

Cardiac autonomic neuropathy in cancer survivors can present in the form of orthostatic hypotension, postural orthostatic tachycardia syndrome, and tachycardia [16]. However, in the current study, the basal HR and BP of ALL patients immediately after chemotherapy were found to be lower as compared to the controls.

From HRV analysis, the significantly lower RMSSD and HFnu, total power, and a significantly higher LFnu and LF-HF ratio, in comparison to controls, suggest cardiovascular autonomic dysfunction in these patients. Cardiac autonomic dysfunction suggests an increase in resting sympathetic and decreased parasympathetic tone. Further, the lower 30:15 ratio and E:I ratio indicate reduced vagal modulation and higher DBP response to IHG indicates increased adrenergic drive. This corroborates findings from the study by Christoffersen et al. that reported cardiac autonomic dysfunction in childhood ALL survivors [7]. Cardiac autonomic neuropathy has been constantly associated with adverse cardiovascular events. The likely mechanism could be chemotherapy-related cardiotoxicity.

Endothelial dysfunction has been a common feature of anti-cancer therapy. However, in ALL patients, endothelial dysfunction in the form of increased baseline sVCAM-1 and sICAM-1 levels in children with ALL have been reported at the time of diagnosis, before the start of therapy, in comparison to healthy controls. The lower viscosity and density of blood are associated with a lower Reynolds number and less stress on endothelium after the reduction of the number of leukocytes. This process triggers inflammation, ultimately contributing to the development of endothelial dysfunction [17]. Additionally, it was reported that immediately after chemotherapy the levels had reduced, but remained slightly higher than control levels. In the current study, the endothelial marker, sVCAM-1, was found to be significantly lower immediately after chemotherapy in comparison to controls. Hs-CRP was also found to be lower immediately after chemotherapy in comparison to controls. sICAM-1 was slightly higher than the control group but was not statistically significant.

At the three-month follow-up after chemotherapy, the cardiovascular autonomic function showed improvement in autonomic function with increased RMSSD, SDNN, total power, HFnu, and decreased LFnu and LF-HF ratio. It was suggestive of improvement of vagal modulation over sympathetic activity. The increased E:I ratio and 30:15 ratio also suggested increased parasympathetic reactivity. The reduction in DBP response to IHG suggested decreased sympathetic modulation. Though improvement was seen in autonomic function, the levels of sVCAM-1, sICAM-1, and hs-CRP remained similar to the values seen immediately after chemotherapy. 

Therefore, this suggests that the cardiac autonomic dysfunction in ALL patients, which was lower as compared to the controls immediately by chemotherapy, improves after three months. Hence, the current study provides an insight into the cardiovascular autonomic modulation, which is a significant determinant of cardiovascular health. Cardiovascular autonomic modulation is one of the lifestyle-modifiable factors. Evidence shows that early non-pharmacological interventions like yoga or regular exercise improve cardiac autonomic function among CVD patients [18]. The improvement in cardiovascular autonomic function and endothelial function three months after chemotherapy gives scope for such intervention, which would further improve their cardiovascular health and provide a better quality of life to ALL survivors.

Limitations of the study

Though improvement in cardiac autonomic function was observed three months after chemotherapy, it was not statistically significant. Also, the effect on endothelial and inflammatory markers three months after chemotherapy was not statistically significant. This could be probably due to the moderate sample size of the study. A study conducted on a larger sample size would enable elucidating changes better.

## Conclusions

We can conclude that chemotherapy has significant implications on cardiovascular autonomic modulation in ALL patients. Cardiovascular autonomic dysfunction manifested as attenuated vagal and increased sympathetic modulation, immediately post chemotherapy as compared to the controls. Three months after chemotherapy, we found an improvement in cardiovascular autonomic modulation. Endothelial function and inflammation were found to be lower compared to the controls immediately after chemotherapy and sustained even three months after chemotherapy. This study further gives an insight that the cardiac autonomic function in these patients can be further improved by adopting appropriate lifestyle interventions.
